# The role of inflammation in myopic retinopathy

**DOI:** 10.3389/fopht.2025.1632047

**Published:** 2025-08-20

**Authors:** Tianxiang Yang, Jinyan Qi, Heping Xu

**Affiliations:** ^1^ Aier Eye Institute, Changsha, China; ^2^ Aier Academy of Ophthalmology, Central South University, Changsha, Hunan, China; ^3^ Wellcome-Wolfson Institute for Experimental Medicine, School of Medicine, Dentistry and Biomedical Sciences, Queen’s University Belfast, Belfast, United Kingdom

**Keywords:** inflammation, pathologic myopia, myopic retinopathy, retinal degeneration, complement system, immune cells

## Abstract

High myopia is a global health concern, often leading to degenerative retinal changes known as myopic retinopathy. Although mechanical stress, hypoperfusion, extracellular matrix remodeling, and growth factor dysregulation have been implicated in the pathogenesis of myopic retinopathy, emerging evidence highlights the critical role of chronic low-grade inflammation. Both innate and adaptive immune systems participate in myopic retinopathy through systemic and local inflammation. Systemically, immune dysregulation is marked by elevated levels of complement proteins C3, autoantibodies anti-LIM and senesce nt cell antigen-like-containing domain protein 1 (anti-LIMS1), and altered circulating immune cells (increased neutrophils and basophils). Locally, retinal homeostasis disruption triggers intraocular inflammation, evidenced by higher levels of interleukin-6 (IL−6), IL−8, tumor necrosis factor α (TNF−α), C-C motif chemokine ligand-2 (CCL2), C−X−C motif chemokine ligand 10 (CXCL10) and activating the complement system. The inflammatory response involves signaling pathways such as JAK-STAT and complement cascades. This review summarizes recent advances in understanding immunological mechanisms underlying myopic retinopathy, offering insights to guide future research.

## 1 Introduction

Myopia is a refractive error where, with accommodation relaxed, parallel light rays focus in front of the retina. This typically results from axial length (axial myopia), but can also stem from excessive refractive power of the cornea or lens (1). Myopia has become a serious global public health concern, particularly in Asia (2–8), driven by lifestyle changes, including reduced time in outdoor activities and prolonged exposure to electronic devices (3). Given current trends, this upward trajectory in myopia prevalence is expected to persist. Epidemiological projections suggest that by 2050, nearly 50% of the global population will be affected by myopia, and approximately 10% will suffer from high myopia (9).

High myopia is associated with multiple degenerative changes within the posterior segment of the eye (10), which may progress to pathologic myopia. According to the International Myopia Institute (IMI), pathologic myopia is defined as excessive axial elongation of the eye associated with myopia, resulting in structural alterations within the posterior segment (1). A key complication is “myopic retinopathy,” encompassing a spectrum of retinal and choroidal changes, including myopic peripheral retinal degeneration (mPRD), posterior staphyloma (PS), leopard-pattern fundus, lacquer cracks, arcuate spots, chorioretinal atrophy, Fuchs spots, myopic rhegmatogenous retinal detachment (mRRD), myopic maculopathy (MM), and myopic choroidal neovascularization (mCNV) (10–12). Myopic retinopathy is influenced by a variety of parameters, including axial length (11, 13), degree of myopia (14), age (14–16), previous ocular disease history (17), choroidal thickness (18), as well as environmental and lifestyle factors (19–22). The pathophysiology of myopic retinopathy is complicated and not completely understood. Potential mechanisms include mechanical stress (11), choroidal hypoperfusion (23), aberrant extracellular matrix remodeling (24), inflammatory and oxidative stress responses (25, 26), dysregulated growth factor expression (27, 28), and dysfunction of the Bruch’s membrane–RPE–photoreceptor complex (29, 30). Currently, there is no effective treatment for this disease.

Immune response is a protective action of the immune system to harmful stimuli, such as pathogens, damaged cells, or irritants, aimed at eliminating the threat and initiating repair. However, when exaggerated or unresolved, it can become dysregulated, leading to tissue damage and pathologies (31). The retina maintains a delicate balance of cellular, metabolic, and structural homeostasis. Disruption of the balance, for example, due to oxygen and nutrient imbalance, waste accumulation, or interstitial fluid dysregulation, can trigger inflammation (32). Although the primary cause of myopic retinopathy is high myopia-mediated structural changes in the eye, growing evidence links inflammation to the onset and progression of retinal complications (33–37). We highlight recent advances in understanding the role of inflammation in myopic retinopathy, offering insights to guide future research.

## 2 Systemic inflammation and myopic retinopathy

The immune system safeguards the body, and its alterations can affect the health of the eye. Accumulating evidence suggests that systemic inflammation may contribute to the development and progression of myopia and myopic retinopathy (34). Studies have shown an increased prevalence of myopia in individuals with inflammatory or autoimmune diseases, such as allergic conjunctivitis, systemic lupus erythematosus, type 1 diabetes, and other chronic inflammatory disorders (14, 34, 38). The neutrophil-to-lymphocyte ratio (NLR) and platelet-to-lymphocyte ratio (PLR) in the peripheral blood of patients with high myopia are significantly increased (39). We reported a higher neutrophil fraction and an elevated NLR, together with lower absolute counts of lymphocytes, eosinophils, and platelets in people with myopic retinopathy (40). We further found that higher levels of circulating basophils are associated with severe myopic retinopathy, such as mCNV (40). This suggests that changes in circulating immune cells are associated with different degrees of myopic retinopathy. As retinal degeneration progresses, microglia are activated, which may recruit circulating immune cells to remove debris (32), and systemic immune activation may affect retinal inflammation.

Apart from immune cells, modified circulating soluble factors were also detected in individuals with myopic retinopathy. For instance, the concentrations of high-sensitivity C-reactive protein (hs-CRP) and complement protein C3 in the peripheral blood were markedly elevated in myopic retinopathy, and C3 may serve as a predictive risk factor for mCNV (41). This indicates that systemic complement activation may play a role in myopic retinopathy. Moreover, it has been documented that serum concentrations of anti-LIM and senescent cell antigen-like-containing domain protein 1 (anti-LIMS1) autoantibodies were markedly increased and significantly associated with the severity of myopic macular degeneration (42).

In addition to alterations in peripheral immune cells and complement proteins, human genetic studies also suggest a role for systemic immune dysregulation in myopic disease. Large CREAM (Consortium for Refractive Error And Myopia) meta–GWAS (genome-wide association study) for myopia mapped the complement regulator *CD55* and the T–cell–related transcription factor *TOX* among risk loci (43). In addition, bidirectional Mendelian–randomization analyses reported that genetically higher circulating IL–1RA and IL–2 are associated with refractive error (44). A GWAS in Chinese populations identified *VIPR2* (which has anti−inflammatory roles in immune cells) as a robust susceptibility locus for high myopia (45). For pathological myopia, a Japanese GWAS study identified *BLID* (a pro−apoptotic regulator in retina) as a risk gene (46), although it was confirmed neither in Chinese cohorts (47) nor the CREAMs myopic macular degeneration analysis (48). Another GWAS identified *LILRB2* (an inhibitory immune receptor on myeloid cells) as a susceptibility gene for pathological myopia (49). Functional studies suggested that LILRB2 overexpression may impair choroidal homeostasis and promote atrophic changes, and the major histocompatibility complex (MHC) pathway was found to be involvement (49). In mCNV, a recent meta-GWAS uncovered a new locus (near *TEX29*/*LINC02337*) as a shared genetic susceptibility with age-related macular degeneration (AMD), along with other risk variants at *CETP* and the *ARMS2* regions (50).

The causal relationship between systemic immune dysregulation and myopic retinopathy remains elusive. When retinal damage becomes extensive or persistent, intraocular clearance mechanisms become inadequate, and this may trigger the recruitment of circulating immune cells to the lesion site to maintain homeostasis. However, if the recruited cells are malfunctional due to genetic predispositions or other systemic inflammatory disorders, they may accelerate retinal pathology and contribute to myopic retinopathy.

## 3 Local ocular inflammation, myopia development, and myopic retinopathy

### 3.1 Local ocular inflammation in myopia development

Excessive axial length elongation in high myopia results from progressive remodeling of the sclera and choroid. It is now recognized that this process is mediated, at least in part, by immune dysregulation (51). Under hypoxic conditions, scleral fibroblasts secrete high levels of IL−6, which activates the TGF−β1/Smad2/matrix metallopeptidase 2 (MMP-2) signaling axis, promoting fibroblast differentiation, apoptosis, and extracellular matrix (ECM) degradation, leading to decreased scleral stiffness and axial length elongation (52, 53). Inflammatory mediators such as IL−1β trigger scleral fibroblast upregulation of cyclooxygenase-2 (COX-2), inducible nitric oxide synthase (iNOS), and various MMPs (54). This inflammatory activation converts scleral fibroblasts into both modulators and amplifiers of local immune responses, creating a feedback loop between structural remodeling and inflammation. In the choroid, a recent study identified two subsets of macrophages: prenatally derived FOLR2^+^ resident macrophages and infiltrating circulating CD14^+^/CD16^+^ monocytes (55). Resident macrophages support lipid handling and vascular maintenance, and their selective depletion precipitates choriocapillaris vasodegeneration and structural collapse. The thinning of the choroid driven by resident macrophage depletion resembles the choroidal attenuation seen in progressive high myopia. The results suggest that dysregulation of choroidal resident macrophages may facilitate the biomechanical and vascular changes that promote axial length elongation (55).

In the mouse model of simple myopia, scleral NLRP3 inflammasome activation is linked with MMP-2 upregulation, scleral matrix remodeling, and myopia progression (56). The C-C motif chemokine ligand-2 (CCL2) can recruit monocytes to differentiate into macrophages, and the infiltrating macrophages express high levels of MMP-2, which promotes myopia progression (57). Other studies have detected M2 macrophages in the sclera during myopia development, and inhibiting M2 macrophages can significantly alleviate myopia progression (58). Collectively, scleral fibroblasts and choroidal macrophages serve as dynamic, inflammation-responsive agents that mediate ECM alteration, vascular remodeling, and axial length elongation in myopia development and progression.

### 3.2 Local ocular inflammation in myopic retinopathy

Ocular immune privilege is maintained through a multi-layered defense system, including (i) physical barriers, primarily the blood–retina barrier (BRB), (ii) immunological and biochemical barriers, including various immunosuppressive molecules secreted by retinal neurons and RPE cells, and (iii) systemic immune regulation, such as the induction of regulatory T cells (Tregs) through mechanisms such as anterior chamber-associated immune deviation (ACAID) (59–61). In addition, the retina actively promotes immune tolerance. Upon encountering retinal antigens, naïve T cells differentiate into antigen-specific Tregs and anergic T cells. The Tregs suppress local autoimmune responses, while anergic T cells become unresponsive to further stimulation, both contributing to immune homeostasis (60, 62–65).

At the early stages of retinal disease, where the BRB remains intact, intraocular inflammatory mediators originate mainly from diseased retinal cells (66). However, if the BRB is damaged, choroidal and circulating immune cells can infiltrate the retina (67). When neurons degenerate, the neuron-immune cross-talk is disrupted and the immunological barrier may fail, leading to dysregulated intraocular inflammation (32). Growing evidence supports the role of ocular inflammation in the pathogenesis of myopic retinopathy, particularly the dysregulation of inflammatory cytokines and complement components.

#### 3.2.1 Inflammatory cytokines and chemokines in myopic retinopathy

Studies from patient-derived samples support the role of ocular inflammation in myopic retinopathy. Higher intraocular levels of inflammatory factors have been detected in eyes with various types of myopic retinopathy. The aqueous humor from mCNV patients contained elevated levels of IL-8 and C−X−C motif chemokine ligand 10 (CXCL10) (68), platelet−derived growth factor (PDGF), IL-2, IL-5, IL-13, IL-15, IL-17A and TNF-α compared to the aqueous humor from simple myopia (69). A meta-analysis reported that the levels of VEGF and IL-8 in the aqueous humor of mCNV patients were higher than those of high myopia without CNV (70). The increasing inflammatory cytokines and chemokines indicate the potential involvement of JAK-STAT, MAPK, PI3K-AKT, and NF−κB signaling in mCNV. Indeed, proteomic analysis of the aqueous humor of mCNV patients showed that, compared with the myopic atrophic maculopathy group and the myopic non-maculopathy group, the differential proteins were significantly enriched in the JAK-STAT signaling pathway (71). Interestingly, apolipoprotein A-1 (APOA1), a protein known for its anti-inflammatory properties and association with chronic inflammation (72), was found to be elevated in the aqueous humor of patients with pathologic myopia (35).

We found that the intraocular inflammatory factors are related to the severity of myopic retinopathy. We investigated the aqueous humor inflammatory factors in patients with different degrees of myopic retinopathy: simple myopia, posterior scleral staphyloma, and posterior scleral staphyloma combined with chorioretinal atrophy. The pro-inflammatory cytokines (Chi3l1, IL-6Rα, IL-8, IL-12, IL-27) and inflammation-related cytokines (A proliferation-inducing ligand (April), B-cell activating factor (BAFF), IL-34) increased, and the anti-inflammation cytokines (IL-11 and aggrecan) decreased progressively with the severity of myopic retinopathy (73). The JAK-STAT signaling pathway was also found to be potentially involved in myopic retinopathy progression (73). Although in-depth studies of the JAK−STAT signaling pathway in myopic retinopathy are limited, it has been explored in other retinal degenerative diseases. Cell−specific STAT3 activation by deleting its inhibitor SOCS3 in rod photoreceptors upregulated anti−apoptotic genes and markedly slowed photoreceptor degeneration and preserved visual function in rd10 and rds mice (74). Transient STAT3 activation in RPE cells curbed oxidative stress; however, when driven chronically by IL−6, it amplified complement factor B (CFB) expression and sterile inflammation (75, 76). Conversely, mice with sustained STAT3 activation across the entire retina develop progressive photoreceptor loss and worsening uveitis, underscoring the neurotoxic potential of the JAK-STAT pathway when left unchecked (77). Thus, proper control of the JAK-STAT3 signaling is neuroprotective, and sustained activation drives inflammatory damage. The JAK−STAT3 signaling pathway may be targeted to control myopic retinopathy.

Altered cytokine levels in other ocular fluids, including the vitreous humor and tears, of patients with high myopia have also been examined. IL-5 and CXCL10 were significantly higher in high myopia and rhegmatogenous retinal detachment (78). Similarly, the levels of CCL2, IL-6, interferon−γ (IFN-γ), eotaxin, macrophage inflammatory protein−1 α (MIP-1α), IL-4, granulocyte−colony–stimulating factor (G-CSF), and CXCL10 in the vitreous of patients with high myopia with macular holes were significantly higher than those in patients with non-high myopia macular holes (78, 79). The levels of IL-6 and CCL2 in the tears from patients with high myopia were significantly increased, and correlated with the severity of myopic maculopathy (80). The authors suggested that these cytokines may be used as biomarkers to predict myopic maculopathy.

#### 3.2.2 The complement system in myopic retinopathy

**Figure 1:**
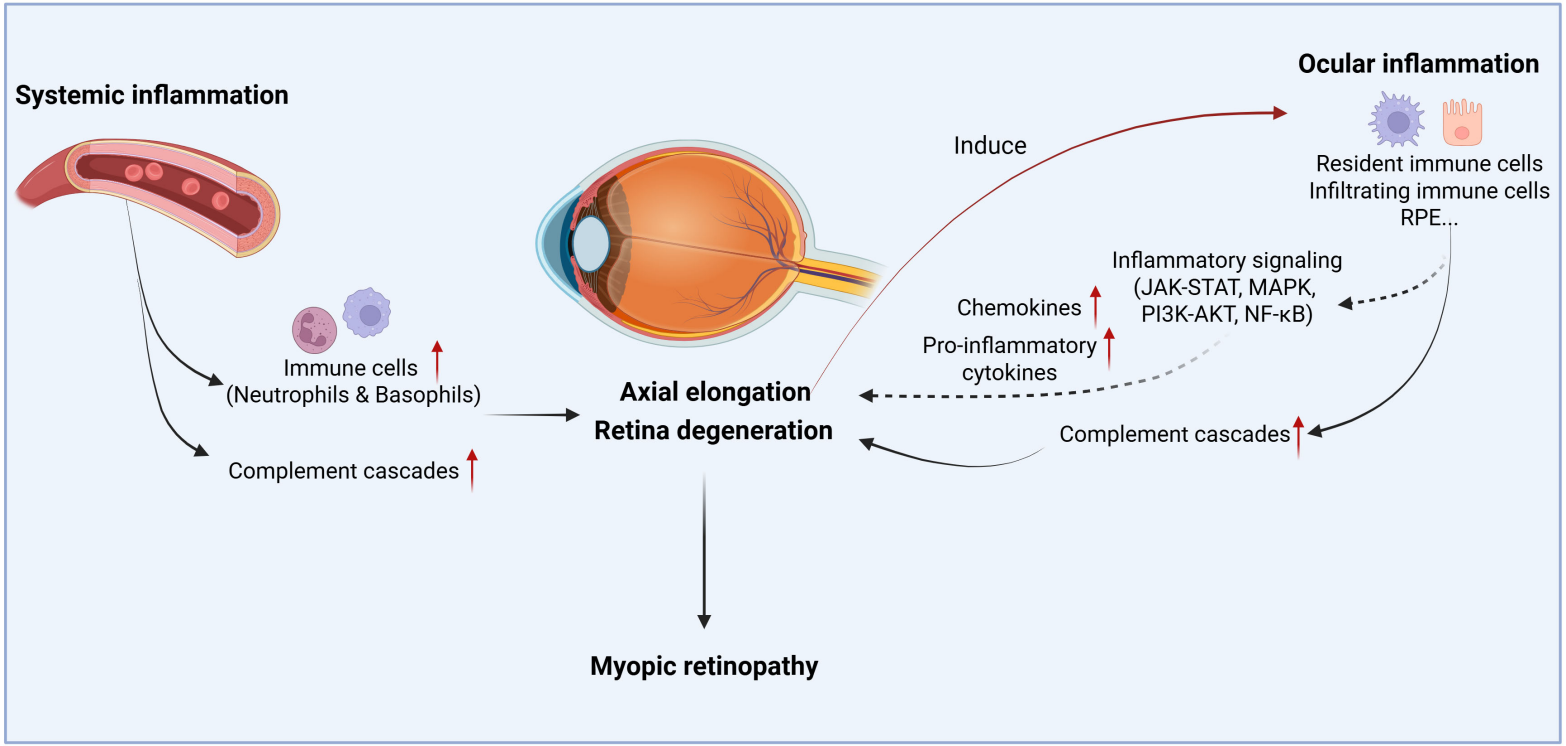
The schematic overview illustrates the role of inflammation in myopic progression and myopic retinopathy. Both systemic and local inflammatory responses participate in the disease progression. Circulating immune cells or inflammatory cytokines/chemokines can infiltrate the eye and participate in scleral remodeling, axial elongation, or retinal degeneration. When retina degenerates from pathological myopia, it induces intraocular inflammation, which in turn, may further promote retinal degeneration. Created with BioRender.com.

The complement system can be activated through the classical pathway, mannan-binding lectin (MBL) pathway, and the alternative pathway (81). The cleavages of C3 and C5 are two critical steps for the full activation of the complement system. The resulting fragments, such as C4b, C3a, C3b, and C5a, drive inflammation by opsonizing dead cells and debris for phagocytic clearance and participating in immune activation (81). The complement system has been implicated in the pathogenesis of myopic retinopathy. In the form-deprivation-induced mild/moderate myopia in guinea pigs, whereby the retina develops peripheral photoreceptor degeneration, we detected significant upregulation of the complement-related genes using RNA-seq technology (82). Higher expression levels of complement genes, such as *C2*, *C3*, and *C4a*, have been reported in other myopic models (83), including the chick FDM model (36) and the guinea pig model of negative lens-induced myopia (84). Proteomic analysis of aqueous humor from patients with pathologic myopia demonstrated prominent involvement of complement and coagulation cascades in disease progression (27). Additionally, our study of 147 myopic patients has shown significantly elevated levels of complement proteins involved in both the classical pathway (C1q, C2, C3, C4, and C4b) and alternative pathway (CFB, CFI, and C3b/iC3b) in the aqueous humor of patients with myopic retinopathy (83). C3b/iC3b and C4 showed a strong negative correlation with retinal neuronal thickness and vascular density in the macula and optic nerve head (83). These results suggest that intraocular complement activation may contribute to retinal vascular and neuronal degeneration in myopic eyes (**Figure 1**).

## 4 Limitations of animal models

Animal models are essential for understanding the mechanism of myopic retinopathy. Several animal models have been reported. Chicks, whose eyelids were sutured for 8 weeks, showed retinal changes similar to lacquer cracks (85). G-protein subunit beta 1 (*GNB3*) gene global knockout chicks showed fundus lacquer cracks in the early stages, which developed into circular lesions with patchy of atrophy at 134 weeks (86). Since the chicken sclera is composed of cartilage and fibrous layers, which are quite different from those of humans (87), these chicken models have not been widely used to study the pathogenesis of myopic retinopathy. The *Lumican*-*Fibromodulin* double knockout mice displayed features of pathological myopia, including scleral thinning and retinal detachment (88). This mouse model is hampered by corneal opacity, systemic connective−tissue abnormalities, and only a modest (~10 %) axial−length increase, making it not an ideal model for human pathological myopia (88). Conditional *Lrp2* knockout mice (KO in neural retina, RPE and ciliary body epithelium) develop retinal thinning and posterior scleral staphylomas (89). Furthermore, the RPE−specific *Lrp2* knockout mice exhibited significant ocular axial length elongation, severe pan−retinal thinning/degeneration with vision loss, and typical RPE anomalies such as macromelanosome formation (90). These phenotypes mirror retinal complications observed in pathological myopia patients (90). This RPE-specific *Lrp2* knockout mouse model may be a useful tool for investigating the mechanism of myopic retinopathy.

## 5 Clinical translation for myopic retinopathy

Standardization and clinical feasibility of detecting inflammatory markers are critical for successful clinical translation. Validating non−invasive biomarkers and incorporating them into early−intervention trials can bridge the gap between mechanistic insights and clinical application. Levels of tear IL−6 and CCL2/MCP−1 have been shown to correlate with axial length and may serve as predictive biomarkers for myopic macular degeneration (80). Moreover, omics-based approaches have identified additional innate−immune signatures, such as intraocular soluble intercellular adhesion molecule 1 (sICAM−1) (91). This biomarker−guided, early−intervention strategy may facilitate the evaluation of anti−inflammatory therapies for myopic retinopathy.

In animal models, various anti-inflammatory agents, such as lactoferrin, diacerein, resveratrol−based botanicals, and the NLRP3 inhibitor MCC950 have been shown to reduce the expression of IL−6, TNF−α, MMP−2 and related mediators, resulting in reduced axial elongation (92–96). Low-concentration of atropine eyedrop, which is widely used for myopic control, also suppresses the expression of c−Fos, IL−6, NF−κB and TNF−α in a hamster model (34). To enhance patient compliance, a self−powered eyelid−activated delivery system for atropine administration has been proposed (97). Interestingly, the immunosuppressive medication cyclosporine A has also demonstrated the ability to slow myopic progression (34). In laser−induced CNV, infliximab reduced retinal oedema (98), while anti−VEGF agents are thought to suppress myopic CNV partly through downregulation of inflammatory cytokines (99). Targeting the JAK−STAT signaling pathway represents another promising therapeutic avenue. Tofacitinib (targets JAK1/3), ruxolitinib (targets JAK1/2), and AG490 (targets JAK2) have demonstrated neuroprotective and anti-angiogenic effects in preclinical models (100–102). Complement-based therapies, including a C3 inhibitor and a C5-targeting aptamer, have been approved by the FDA for treating geographic atrophy type of AMD (103) and may be repurposed for the management of myopic retinopathy.

## 6 Summary

Myopic retinopathy is associated with changes in the immune system, both systemically and locally within the intraocular microenvironment (**Figure 1**). When retinal degeneration develops, ocular immune privilege may be compromised and circulating immune cells such as monocytes, neutrophils, and soluble factors, including complement proteins, may infiltrate the retina, leading to dysregulated intraocular inflammation, which may further promote retinal degeneration (**Figure 1**).

Emerging evidence suggests a bidirectional relationship between chronic inflammation and myopic retinal degeneration (33) (**Figure 1**). On one hand, a pro-inflammatory microenvironment may exacerbate myopia progression. Epidemiological data show that children with systemic inflammatory diseases have significantly higher rates of myopia compared to healthy controls (34). In experimental models, induced inflammation accelerates pathologic axial elongation, whereas anti-inflammatory interventions mitigate myopic eye growth (34). On the other hand, high myopia-mediated retinal degeneration can, in turn, trigger intraocular inflammation. Pathologic myopic eyes exhibit elevated levels of inflammatory cytokines and complement components, which correlate with the extent of retinal thinning, indicating that more severe myopic retinal degeneration is associated with greater inflammatory activities (83, 104).

Therefore, inflammation may not be the primary initiator of myopic retinopathy, retinal degeneration disrupts immune privilege and precipitates inflammatory cascades that further accelerate tissue damage. Controlling inflammation, along with conventional strategies to limit axial elongation, may offer a dual approach to slow the progression of myopic retinopathy.

## 7 Future directions

Despite significant progress in understanding the role of inflammation in myopic retinopathy, critical knowledge gaps remain. Clinical research is unlikely to definitively establish the causal role of inflammation or fully elucidate the underlying mechanisms due to confounding variables and the chronic nature of disease progression. Meanwhile, basic science research is hindered by the lack of reliable animal models that accurately recapitulate the pathological features of myopic retinopathy. Future investigations should prioritize the development of physiologically relevant models of pathological myopia or myopic retinopathy, including human induced pluripotent stem cells (iPSCs)-derived ocular organoids or animal models, delineation of the cellular sources and regulatory pathways of inflammation, and clarification of the precise role of inflammation in the onset and progression of myopic retinal degeneration. It also remains uncertain whether targeted anti−inflammatory therapies can effectively prevent or reverse the course of the disease. Addressing these challenges will be essential for advancing our understanding of the pathogenesis of myopic retinopathy and for developing effective therapeutic strategies.
